# Compressive Wideband Spectrum Sensing Aided Intelligence Transmitter Design

**DOI:** 10.3390/s25082400

**Published:** 2025-04-10

**Authors:** Lizhi Qin, Yuming Chen, Leli Zhong, Hongzhi Zhao

**Affiliations:** National Key Laboratory of Wireless Communications, University of Electronic Science and Technology of China, Chengdu 611731, China; 202312220625@std.uestc.edu.cn (L.Q.); 202322220426@std.uestc.edu.cn (Y.C.); 2021190501015@std.uestc.edu.cn (L.Z.)

**Keywords:** nyquist folding receiver, interference cancellation, compressive sensing, time synchronization errors

## Abstract

In order to realize robust communication in complicated interference electromagnetic environments, an intelligent transmitter design is proposed in this paper, where an auxiliary wideband receiver senses the electromagnetic distribution information in a wide bandwidth range to decide the optimal working frequency. One of the key issues is suppressing the self-interference of high-power transmitter signals to the co-platform wideband sensing receiver. Due to the multipath effect of the self-interference channel, perfect time synchronization of self-interference signals is not achievable, which reduces the interference cancelation performance of the co-platform. Therefore, this paper investigates the impact of time synchronization errors on the self-interference cancellation performance of the Nyquist folding receiver (NYFR)-based system. First, a self-interference cancellation architecture based on NYFR is proposed to support the realization of real-time wideband spectrum sensing. Secondly, closed-form expressions for the residual interference power and the self-interference cancellation performance are derived, and the impact of reference signal sampling errors on the self-interference cancellation performance is also analyzed. Theoretical analysis and simulation results show that the NYFR-based self-interference cancellation performance decreases with increasing time synchronization errors and folding multiples, and the system is especially sensitive to time synchronization errors. Moreover, frequency detection simulations show that, under an SI-to-NCS power ratio of 0 dB, the proposed interference cancellation scheme improves the frequency detection probability by approximately 80%. The research results provide a theoretical reference for the compressed sensing-aided intelligent transmitter realization.

## 1. Introduction

Due to the ubiquitous and broadcast nature of electromagnetic waves in wireless networks, communication platforms are highly susceptible to interference from non-cooperative signals during transmission. Ensuring the security and reliability of these platforms is therefore a critical challenge [1,2]. Effective interference sensing and decision-making play a vital role in enhancing communication security and improving the anti-interference capability of transmitted signals [3]. These processes involve extracting key characteristics of interference signals and making intelligent decisions regarding essential transmission parameters such as frequency and bandwidth [4]. To address this challenge, interference sensing must be seamlessly integrated into the communication system, an issue that has garnered significant attention in recent research by the Third Generation Partnership Project (3GPP) [5]. During the interference sensing phase, the receiver must monitor the broadest possible frequency range [6], as the frequency range of interference signals is typically unknown [7].

Conventional wideband sensing methods require high-precision analog-to-digital converters (ADCs) to achieve accurate signal sampling [8]. To address the trade-off between wideband sensing requirements and the limited sampling rate of ADCs, Fudge proposed the Nyquist Folding Receiver (NYFR) based on compressed sensing (CS) theory [9]. This approach enables the digital acquisition of sparse or compressible wideband signals at sub-Nyquist sampling rates. Furthermore, these signals can be analyzed using time-frequency signal processing techniques to extract relevant information based on their inherent characteristics. Existing research has explored various aspects of spectrum sensing [10], modulation classification [11], and modulated signal detection [12].

However, during the NYFR-based sensing process, the high transmission power of the transmitter induces significant self-interference to the NYFR. This interference prevents the sensing receiver from functioning properly [13]. Therefore, it is crucial for the NYFR to effectively mitigate self-interference under the constraints of limited space [14]. Existing research primarily focuses on self-interference cancellation methods and performance analysis for conventional receivers [15,16]. However, due to hardware limitations, non-ideal factors can adversely affect the performance of these techniques [17]. For instance, Guo et al. [18,19] investigated the adverse effects of imperfect time synchronization and sampling clock offset on interference cancellation performance. Additionally, He et al. [20] analyzed the impact of time alignment errors on nonlinear interference cancellation. Furthermore, existing studies have examined the impact of frequency offset on adjacent channel interference suppression and transceiver optimization for secure communication systems under frequency mismatches [21,22]. It is worth noting that these studies are based on conventional receivers. To the best of our knowledge, self-interference cancellation studies for NYFR have yet to be reported.

In this paper, a CS-aided intelligent transmitter is proposed, which utilizes an NYFR-based receiver to sense non-cooperative signals and perform self-interference cancellation. Furthermore, the impact of time synchronization errors on interference cancellation performance is analyzed. The main contributions of this work are summarized as follows:

- An NYFR-based self-interference cancellation architecture is proposed to support real-time wideband spectrum sensing in intelligent transmitters, addressing the challenge of co-platform interference suppression under complex electromagnetic environments.
- Closed-form analytical expressions for the residual interference power and cancellation performance are derived, taking into account the impact of time synchronization errors and sampling inaccuracies, which quantitatively reveal the performance limitations of the proposed scheme.
- Theoretical analysis and simulation results demonstrate the effectiveness of the proposed method. Specifically, it is shown that the interference cancellation performance degrades with increasing synchronization errors and folding factors. Moreover, simulation results verify that, at an SI-to-NCS power ratio of 0 dB, the proposed scheme improves the frequency detection probability by approximately 80%.

The structure of this paper is outlined as follows: Section 2 introduces the system model. Section 3 analyzes the impact of time synchronization errors on the self-interference cancellation performance of a wideband sensing receiver. Section 4 provides simulation results to validate the analysis. Finally, Section 5 concludes the paper.

## 2. System Model

**Figure 1:**
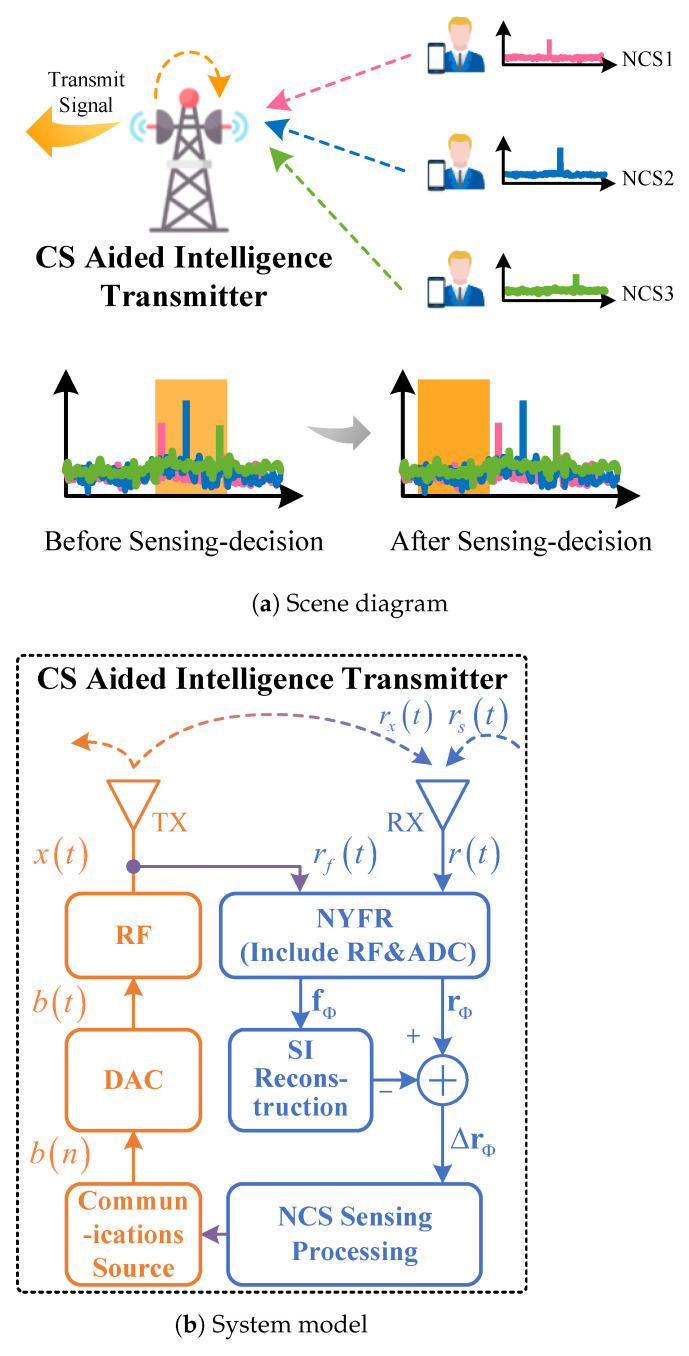
The CS aided intelligent transmitter.

As illustrated in Figure 1a, the CS-aided intelligent transmitter accurately avoids non-cooperative signals (NCS) by sensing NCS1, NCS2, and NCS3 and dynamically adjusting its operating frequency. In contrast, conventional transmitters lack the capability to effectively detect and adapt to non-cooperative signals.

Figure 1 shows the CS-aided intelligent transmitter scene diagram and system model, where NYFR receives both self-interference signal (SI) $r_{x}\left(t\right)$ and NCS $r_{s}\left(t\right)$ from the surrounding complex electromagnetic environment.

To mitigate the impact of SI, the transmit signal $x\left(t\right)$ is wired and connected to the wideband sensing receiver as a reference signal $r_{f}\left(t\right)$. Subsequently, the received signal $r\left(t\right)$ and the reference signal are folded, respectively, and the folded reference signal $f_{\Phi }$ is used to reconstruct and cancel the folded received signal $r_{\Phi }$.

The CS-aided intelligent transmitter generates a baseband signal $b\left(n\right)$, which is then converted to an analog signal $b\left(t\right)$ by digital-to-analog conversion (DAC) and subsequently upconverted to a radio frequency (RF) signal $x\left(t\right)$

(1) $$\begin{array}{c} x\left(t\right)=b\left(t\right)e^{j\left[2\pi f_{t}t+\theta_{t}\right]}, \end{array}$$

where $f_{t}$ and $\theta_{t}$ are the carrier frequency and initial phase, respectively.

The $x\left(t\right)$ causes self-interference to the wideband sensing receiver. The channel is modeled as a multipath due to SI reflections from objects such as the vehicle platform, aircraft, and buildings. Consequently, the SI is expressed as

(2) $$\begin{array}{c} r_{x}\left(t\right)=\sum_{l=1}^{L}h_{l}x\left(t-\tau_{l}\right), \end{array}$$

where *L* denotes the number of multipath, $h_{l}$ denotes the complex channel gain of the *l*-th path, and $\tau_{l}$ denotes the relative delay between the *l*-th path of the SI and the reference signal.

The SI is wired to the sensing receiver as a reference signal $r_{f}\left(t\right)=x\left(t\right)$.

In the transmitter, the sensing receiver receives a signal $r\left(t\right)$ that is expressed as

(3) $$\begin{array}{c} r\left(t\right)=r_{x}\left(t\right)+r_{s}\left(t\right)+z\left(t\right), \end{array}$$

where $r_{s}\left(t\right)$ is the NCS, $z\left(t\right)$ is the additive white Gaussian noise and obeys a zero-mean Gaussian distribution.

After down-conversion and analog-to-digital conversion, the reference signal is expressed as $r_{f}\left(n\right)=b\left(n\right)$, and the received signal is expressed as

(4) $$\begin{array}{c} r\left(n\right)=r_{x}\left(n\right)+r_{s}\left(n\right)+z\left(n\right), \end{array}$$

The self-interference signals $r_{x}\left(n\right)$ are

(5) $$\begin{array}{c} r_{x}\left(n\right)=\sum_{l=1}^{L}h_{l}b\left(n-D_{l}\right), \end{array}$$

where Dl=τlτlTT represents the relative time delay between the *l*-th path and the reference signal, with *T* denoting the sampling period.

## 3. NYFR-Based Self-Interference Cancellation Performance Analysis

Due to imperfect time synchronization, complete elimination of the self-interference signal is unattainable. The combined effects of time synchronization errors and NYFR on SI cancellation performance are analyzed in detail.

### 3.1. NYFR-Based Signal Folding

The received signal $r\left(n\right)$ and the reference signal $r_{f}\left(n\right)$ at the NYFR input are each represented N-dimensional vectors of length

(6) $$\begin{array}{c} r={\left[r\left(n+1\right),r\left(n+2\right),\ldots ,r\left(n+N\right)\right]}^{T}, \end{array}$$

(7) $$\begin{array}{c} f={\left[r_{f}\left(n+1\right),r_{f}\left(n+2\right),\ldots ,r_{f}\left(n+N\right)\right]}^{T}. \end{array}$$

After NYFR, the received signal $r_{\Phi }$ and the reference signal $f_{\Phi }$ can be represented separately as vectors of length *K*.

(8) $$\begin{array}{cc} r_{\Phi } & =\Phi r=\Phi r_{x}+\Phi r_{s}+\Phi z\\ \; & ={\left[r\left(n+\gamma_{1}\right),r\left(n+\gamma_{2}\right),\ldots ,r\left(n+\gamma_{K}\right)\right]}^{T}, \end{array}$$

(9) $$\begin{array}{cc} f_{\Phi } & =\Phi f\\ \; & ={\left[r_{f}\left(n+\gamma_{1}\right),r_{f}\left(n+\gamma_{2}\right),\ldots ,r_{f}\left(n+\gamma_{K}\right)\right]}^{T}, \end{array}$$

where $\gamma =\left[\gamma_{1},\gamma_{2},\ldots ,\gamma_{K}\right]$ is the index vector of Φ, and Φ is the measurement matrix

(10) $$\begin{array}{c} \Phi =\left[\begin{array}{ccc} \phi_{1,1} & \cdots & \phi_{1,N}\\ \vdots & \ddots & \vdots \\ \phi_{K,1} & \cdots & \phi_{K,N} \end{array}\right], \end{array}$$

where $\phi_{k,\gamma_{k}}$ is 1, k=1,2,…,K, $1\leq \gamma_{k}\leq N$, the remaining elements are 0.

### 3.2. Time Synchronization Errors

The wideband sensing receiver is assumed to be capable of estimating the channel gain. However, non-ideal factors such as multipath and time synchronization errors can degrade the interference cancellation performance during implementation on vehicle platforms. Therefore, this section focuses on analyzing the impact of time synchronization errors on interference cancellation performance in multipath environments. Assuming that the time delay of the *l*-th path is estimated as

(11) $$\begin{array}{c} {\hat{D}}_{l}=D_{l}+\Gamma_{l}. \end{array}$$

After the time synchronization process, the *l*-th path of the SI experiences a relative time delay error $\Gamma_{l}$$\left(0\leq \left|\Gamma_{l}\right|\leq 0.5\right)$ relative to the reference signal $r_{f}\left(n\right)$, from which an ideal fractional time delay filter can be represent as [23]

(12) $$\begin{array}{cc} {\hat{r}}_{x}^{l}\left(n\right) & =r_{f}\left(n+\Gamma_{l}\right)\\ \; & =r_{f}\left(n\right)*\mathrm{sinc}\left(n+\Gamma_{l}\right)\\ \; & =A_{\Gamma_{l}}r_{f}\left(n\right)+f_{\Gamma_{l}}\left(n\right), \end{array}$$

where $A_{\Gamma_{l}}=\mathrm{sinc}\left(\Gamma_{l}\right)$ denotes the attenuation coefficient due to the time synchronization errors, and $f_{\Gamma_{l}}\left(n\right)$ represents the inter-symbol interference (ISI) noise from the other symbols. Furthermore, $f_{\Gamma_{l}}\left(n\right)$ is uncorrelated with $r_{f}\left(n\right)$, meaning that the ISI noise cannot be cancelled.

(13) fΓln=∑i=−∞i≠0+∞rfn−isinci−Γl.

After time synchronization, the SI can be represented as

(14) $$\begin{array}{cc} {\hat{r}}_{x}\left(n\right) & =\sum_{l=1}^{L}h_{l}{\hat{r}}_{x}^{l}\left(n\right)=\sum_{l=1}^{L}h_{l}r_{f}\left(n+\Gamma_{l}\right). \end{array}$$

The wideband sensing receiver reconstructs the SI as ${\hat{r}}_{f}\left(n\right)=\sum_{l=1}^{L}h_{l}A_{\Gamma_{l}}r_{f}\left(n\right)$.

If the receiver is not folded, the vector forms of the time-synchronized SI ${\hat{r}}_{x}$ and the reconstructed SI $\hat{f}$ is expressed as

(15) $$\begin{array}{c} {\hat{r}}_{x}={\left[{\hat{r}}_{x}\left(n+1\right),{\hat{r}}_{x}\left(n+2\right),\ldots ,{\hat{r}}_{x}\left(n+N\right)\right]}^{T}, \end{array}$$

(16) $$\begin{array}{c} \hat{f}={\left[{\hat{r}}_{f}\left(n+1\right),{\hat{r}}_{f}\left(n+2\right),\ldots ,{\hat{r}}_{f}\left(n+N\right)\right]}^{T}. \end{array}$$

After the receiver folds the received signal, the time-synchronized SI ${\hat{r}}_{\Phi }^{x}$ and the reconstructed SI ${\hat{f}}_{\Phi }$ is expressed as

(17) $$\begin{array}{c} {\hat{r}}_{\Phi }^{x}={\left[{\hat{r}}_{x}\left(n+\gamma_{1}\right),{\hat{r}}_{x}\left(n+\gamma_{2}\right),\ldots ,{\hat{r}}_{x}\left(n+\gamma_{K}\right)\right]}^{T}, \end{array}$$

(18) $$\begin{array}{c} {\hat{f}}_{\Phi }={\left[{\hat{r}}_{f}\left(n+\gamma_{1}\right),{\hat{r}}_{f}\left(n+\gamma_{2}\right),\ldots ,{\hat{r}}_{f}\left(n+\gamma_{K}\right)\right]}^{T}. \end{array}$$

### 3.3. Effects on Self-Interference Cancellation Performance

Define the self-interference cancellation performance *G* as

(19) $$\begin{array}{cc} G & =10\mathrm{lg}\frac{P\left\{{\hat{r}}_{\Phi }^{x}\right\}+P\left\{\Phi z\right\}}{P\left\{\Delta r_{\Phi }^{x}\right\}+P\left\{\Phi z\right\}}\;, \end{array}$$

where $P\left\{\cdot \right\}$ is the power of $\left\{\cdot \right\}$.

Assuming that the baseband signal $b\left(n\right)$ follows a Gaussian distribution, the power of $b\left(n+\Gamma_{l}\right)$ is

(20) $$\begin{array}{c} P\left\{b\left(n+\Gamma_{l}\right)\right\}\approx P\left\{b\left(n\right)\right\}. \end{array}$$

Thus, the power of ${\hat{r}}_{\Phi }^{x}$ is expressed as

(21) $$\begin{array}{cc} P\left\{{\hat{r}}_{\Phi }^{x}\right\} & =P\left\{\Phi \left[{\hat{r}}_{x}\left(n+1\right),\ldots ,{\hat{r}}_{x}\left(n+N\right)\right]\right\}\\ \; & =P\left\{\sum_{l=1}^{L}h_{l}b\left(n+\Gamma_{l}\right)\right\}\approx P\left\{b\left(n\right)\right\}\sum_{l=1}^{L}{\left|h_{l}\right|}^{2}. \end{array}$$

Additionally, it is assumed that the power of the Gaussian noise Φz is denoted by $\sigma^{2}$. Since the noise is independent of the SI, the power of the received SI plus noise can be computed using the following equation in corporation with Equation (19).

(22) $$\begin{array}{cc} P\left\{{\hat{r}}_{\Phi }^{x}\right\}+P\left\{\Phi z\right\} & =P\left\{b\left(n\right)\right\}\sum_{l=1}^{L}{\left|h_{l}\right|}^{2}+\sigma^{2}. \end{array}$$

The power of $f_{\Gamma_{l}}\left(n\right)$ is expressed as

(23) PfΓln=Prfn∑i=−∞i≠0∞sinci−Γl2.

The residual SI $\Delta r_{\Phi }^{x}$ is expressed as Equation (24) with its power given by Equation (25), and ${ℜ}_{l}$ denotes the ISI.

(24) $$\begin{array}{cc} \Delta r_{\Phi }^{x} & ={\hat{r}}_{\Phi }^{x}-{\hat{f}}_{\Phi }=\Phi \left[{\hat{r}}_{x}-\hat{f}\right]\\ \; & =\Phi {\left[\begin{array}{c} \sum_{l=1}^{L}h_{l}f_{\Gamma_{l}}\left(n+1\right),\ldots ,\sum_{l=1}^{L}h_{l}f_{\Gamma_{l}}\left(n+N\right) \end{array}\right]}^{T}. \end{array}$$

(25) PΔrΦx=PΦ∑l=1LhlfΓln+1,…,∑l=1LhlfΓln+N=PΦbn+1∑l=1Lhl∑i=−∞i≠0∞sinci−Γl,…,bn+N∑l=1Lhl∑i=−∞i≠0∞sinci−Γl≈Pbn∑l=1Lhl2∑i∈γsinci−Γl2+∑i∈γ−Nsinci−Γl2=Pbn∑l=1Lhl2ℜl.

(26) $$\begin{array}{c} {ℜ}_{l}=\sum_{i\in \gamma }\mathrm{sinc}{\left(i-\Gamma_{l}\right)}^{2}+\sum_{i\in \gamma -N}\mathrm{sinc}{\left(i-\Gamma_{l}\right)}^{2}. \end{array}$$

The power of the residual SI plus noise in Equation (19) using the following equation

(27) $$\begin{array}{cc} P\left\{\Delta r_{\Phi }^{x}\right\}+P\left\{\Phi z\right\} & =P\left\{b\left(n\right)\right\}\sum_{l=1}^{L}{\left|h_{l}\right|}^{2}{ℜ}_{l}+\sigma^{2}. \end{array}$$

Thus, the self-interference cancellation performance is obtained.

(28) $$\begin{array}{c} G=10\mathrm{lg}\frac{\mathrm{INR}\sum_{l=1}^{L}{\left|h_{l}\right|}^{2}+1}{\mathrm{INR}\sum_{l=1}^{L}{\left|h_{l}\right|}^{2}{ℜ}_{l}+1}, \end{array}$$

where INR=Pbnbnσ2σ2 denotes the SI to noise power ratio.

The above analysis shows that the INR, time synchronization errors, and ISI affect interference cancellation performance. An increase in time synchronization errors results in decreased interference rejection performance. According to the analysis, interference cancellation does not influence the signal’s sparsity or reconstruction probability.

Therefore, the folded NCS can be recovered using traditional CS reconstruction algorithms, or alternative algorithms can be used to extract the information directly [24]. After extracting the useful information from NCS, intelligent decisions can be made regarding the frequency and bandwidth of the transmitted signal to avoid NCS interference. This paper focuses on interference cancellation performance, leaving signal reconstruction aspects beyond its scope.

## 4. Numerical Simulations

Most radar attack systems currently operate within a frequency range of 2∼18 GHz, where jamming signals satisfy the frequency domain sparsity within this range. For simulation purposes, the frequency range is reduced proportionally from 16 GHz to 100 MHz, and the bandwidth for the SI is set to 1 MHz. Simulations are performed using the MATLAB 2022b tool to validate the accuracy of the wideband sensing receiver’s self-interference cancellation performance.

**Figure 2:**
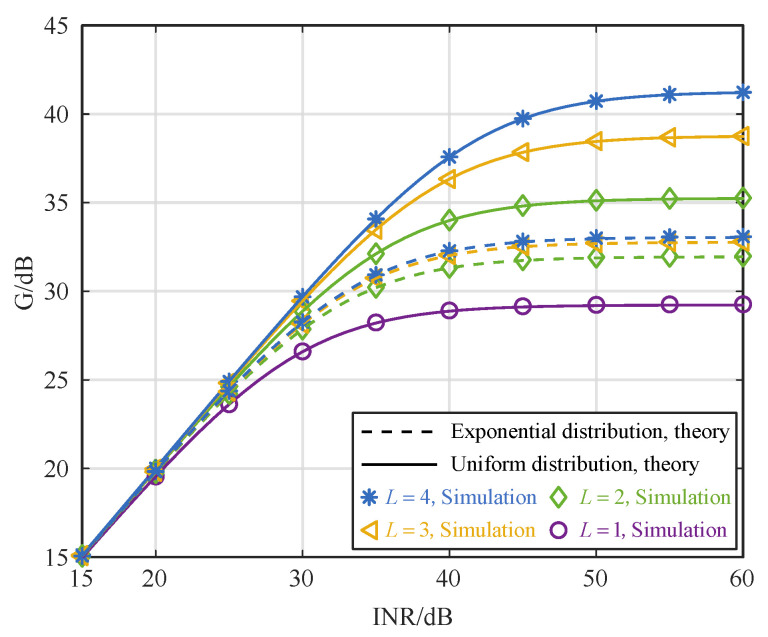
Relationship between interference cancellation performance and INR with 0.5T time synchronization errors.

When the folding mode is $\gamma =\left[\begin{array}{ccccc} 0 & 2 & 3 & 5 & 7 \end{array}\right]$, and the time synchronization error is 0.5*T*, Figure 2 shows the impact of INR on the interference cancellation performance. The results approximate the theoretical value. When the INR is 15 dB, the interference cancellation performance is also 15 dB, and the SI can be concealed in the noise vicinity. At this point, the impact of time synchronization errors on interference cancellation performance is minimal and can be ignored. When the INR is 15∼40 dB, the interference cancellation performance gradually increases with the increase of INR, and it is simultaneously impacted by noise and time synchronization errors. The impact of the time synchronization errors on interference cancellation performance increases as the INR increases. When the INR is 40∼60 dB, the interference cancellation performance remains almost unchanged with an increase in INR. It shows that time synchronization errors are the main factor affecting interference cancellation performance.

**Figure 3:**
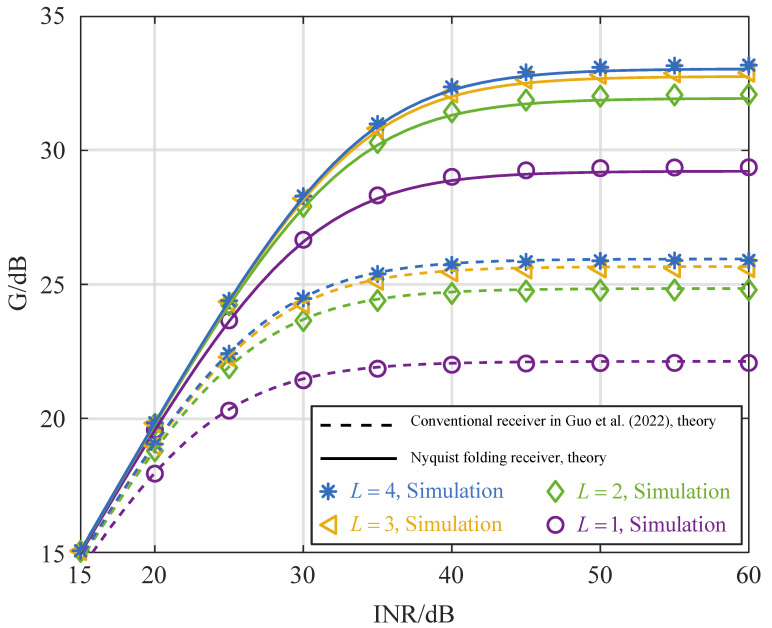
Interference cancellation performance of NYFR and conventional receiver at 0.5T time synchronization errors [19].

Based on Figure 2, the interference cancellation performance of both the NYFR and the conventional receiver is evaluated through simulations. As shown in Figure 3, the simulation results closely align with the theoretical analysis, validating the accuracy of the proposed model. It can be observed that the interference cancellation performance of both receivers improves as the INR increases, eventually reaching saturation. Notably, when the INR exceeds 40 dB, the NYFR achieves approximately 7 dB higher interference cancellation performance compared to conventional receivers. When the ISI characterized by Equation (26) is incorporated into the interference cancellation process of a conventional receiver, the index *i* ranges from −N to *N*. As a result, the value of ${ℜ}_{l}$ increases, leading to a larger non-cancellable interference component, which in turn degrades the overall interference cancellation performance.

The comparison of the interference cancellation performance between the solid and dotted lines in the figure indicates that the variation decreases as the number of multipaths increases. In a scenario where multipath amplitude is uniformly distributed, the interference cancellation performance is influenced by the number of multipath signals present. With the exponential distribution of multipath amplitude, data with multipath numbers 3 and 4 are closer to each other, indicating that the interference cancellation performance is mainly affected by the time synchronization errors.

**Figure 4:**
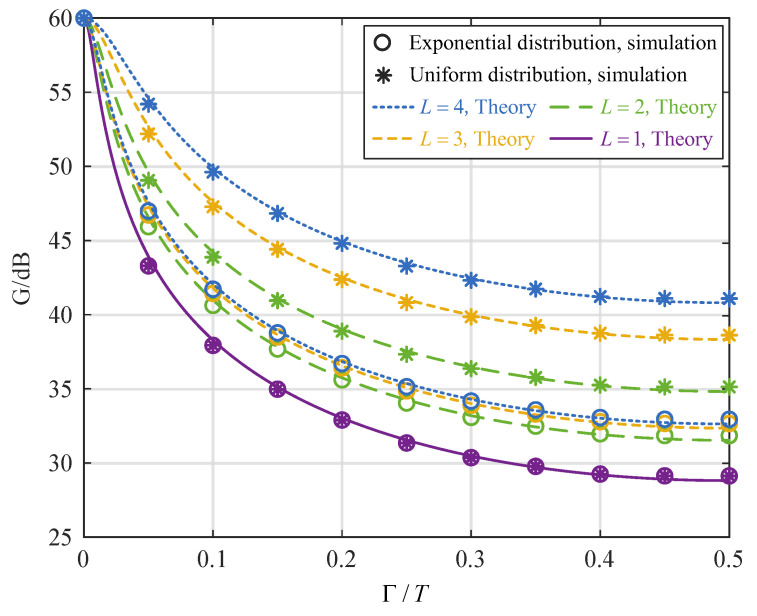
Relationship between interference cancellation performance and time synchronization errors for different numbers of multipaths.

Figure 4 illustrates the impact of time synchronization errors on the interference cancellation performance when the INR is 60 dB, the folding mode $\gamma =\left[\begin{array}{ccccc} 0 & 2 & 3 & 5 & 7 \end{array}\right]$, and the number of multipaths is different. The figure reveals that when the time synchronization error is 0, the interference can be canceled to the noise level, which is consistent with the theoretical value. However, as the time synchronization errors increase, the interference cancellation performance decreases initially and stabilizes. Furthermore, as the number of multipaths increases, the interference cancellation becomes less sensitive to time synchronization errors. Notably, the impact of time synchronization errors is smaller when the multipath amplitudes are uniformly distributed than when they follow an exponential distribution.

**Figure 5:**
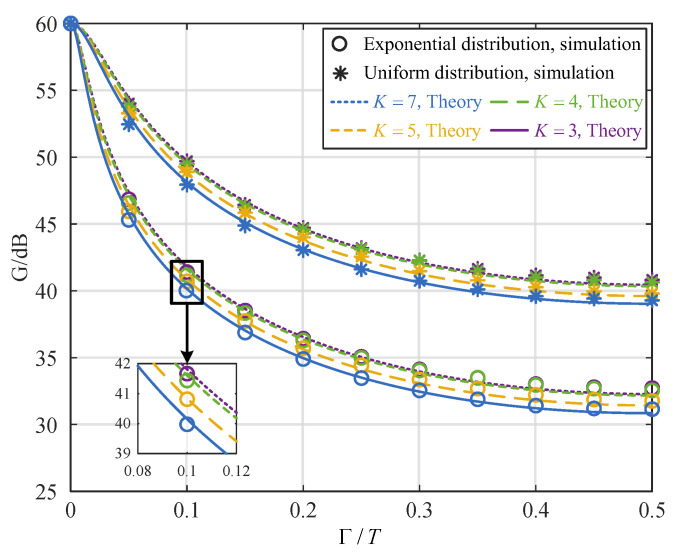
Relationship between interference cancellation performance and time synchronization errors for different folding multiples.

Figure 5 illustrates the impact of time synchronization errors on interference cancellation performance under an INR of 60 dB, four multipaths, and varying folding multiples. The figure shows that when the multipath amplitude distribution is the same, the interference cancellation performance trend is consistent as the time synchronization errors increase. The figure indicates that the interference cancellation performance follows a consistent trend for the same multipath amplitude distribution as the time synchronization errors increase. As the time synchronization errors surpass 0, the interference cancellation performance decreases as the number of *K* decreases and the folding multipliers N/K increase. When the time synchronization error is 0.1*T*, the interference cancellation performance of the 10/7 folding multiple is 1 dB higher than that of the 10/3 folding multiple.

**Figure 6:**
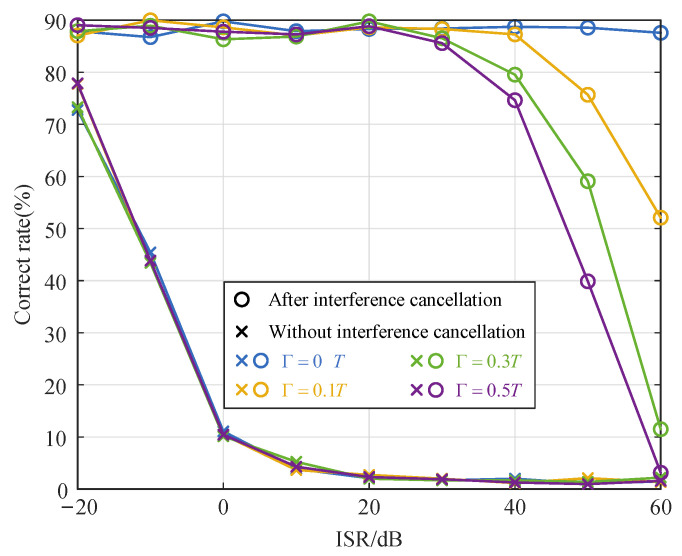
Relationship between frequency estimation correct rate and ISR.

Figure 6 illustrates the relationship between the correct rate of NYFR frequency estimation and the SI-to-NCS power ratio (ISR) under different time synchronization errors. The figure shows that the frequency estimation correct rate is lower when the receiver is without interference cancellation than after interference cancellation. The frequency estimated correct rate decreases rapidly when the ISR is −20∼0 dB. When the ISR exceeds 0 dB, the frequency estimation correct rate gradually decreases from 10% to near 0. After interference cancellation, the frequency estimated correct rate is significantly higher. When the ISR is −20∼20 dB, the frequency estimation correct rate stays near 89%, and when the ISR is greater than 20 dB, the frequency estimation correct rate decreases as the time synchronization errors increase. When the ISR is 60 dB and the time synchronization error is 0.5*T*, the frequency estimation correct rate decreases to about 3%. This shows that as the ISR increases, the time synchronization errors affect the interference suppression performance and thus reduce the frequency estimation correct rate.

## 5. Conclusions

In this paper, a CS-aided intelligent transmitter was proposed to enable interference-robust signal transmission in complex electromagnetic environments. By using an NYFR-based receiver, the design reduces the sampling rate and cost while ensuring continuous wideband spectrum sensing. Furthermore, a closed-form expression for the residual SI power under multipath conditions was derived, revealing the relationship between time synchronization errors and interference cancellation performance. Both theoretical analysis and simulation results demonstrate that the SI cancellation performance deteriorates with increasing time synchronization errors and folding factors. Notably, simulation results show that, under an ISR of 0 dB, the proposed scheme achieves an 80% improvement in frequency detection probability. Future work will focus on validating the proposed scheme through hardware implementation.

## Data Availability

No new data were created or analyzed in this study. Data sharing is not applicable to this article.

## Abbreviations

The following abbreviations are used in this manuscript:

CS	Compressed sensing
NYFR	Nyquist folding receiver
3GPP	Third Generation Partnership Project
ADC	Analog-to-digital converter
NCS	Non-cooperative signal
SI	Self-interference signal
DAC	Digital-to-analog converter
RF	Radio-frequency
ISI	Inter-symbol interference
INR	SI to noise power ratio
ISR	SI to NCS power ratio
